# Vision affects tactile target and distractor processing even when space is task-irrelevant

**DOI:** 10.3389/fpsyg.2014.00084

**Published:** 2014-02-06

**Authors:** Ann-Katrin Wesslein, Charles Spence, Christian Frings

**Affiliations:** ^1^Cognitive Psychology, Department of Psychology, University of TrierTrier, Germany; ^2^Department of Experimental Psychology, University of OxfordOxford, UK

**Keywords:** touch, multisensory integration, selective attention, distractor processing, visuo-tactile interaction

## Abstract

The human brain is adapted to integrate the information from multiple sensory modalities into coherent, robust representations of the objects and events in the external world. A large body of empirical research has demonstrated the ubiquitous nature of the interactions that take place between vision and touch, with the former typically dominating over the latter. Many studies have investigated the influence of visual stimuli on the processing of tactile stimuli (and vice versa). Other studies, meanwhile, have investigated the effect of directing a participant’s gaze either toward or else away from the body-part receiving the target tactile stimulation. Other studies, by contrast, have compared performance in those conditions in which the participant’s eyes have been open versus closed. We start by reviewing the research that has been published to date demonstrating the influence of vision on the processing of tactile *targets*, that is, on those stimuli that have to be attended or responded to. We outline that many – but not all – of the visuotactile interactions that have been observed to date may be attributable to the direction of spatial attention. We then move on to focus on the crossmodal influence of vision, as well as of the direction of gaze, on the processing of tactile *distractors*. We highlight the results of those studies demonstrating the influence of vision, rather than gaze direction (i.e., the direction of overt spatial attention), on tactile distractor processing (e.g., tactile variants of the negative-priming or flanker task). The conclusion is that no matter how vision of a tactile distractor is engaged, the result would appear to be the same, namely that tactile distractors are processed more thoroughly.

## INTRODUCTION

At each and every waking moment, our brains are likely to be processing some combination of visual, auditory, tactile, and even smell stimuli. That said, we are nevertheless able to focus our attention on a single sensory modality at a time, such as on audition when listening to a concert, or on vision when reading a book. However, no less remarkably, we can also integrate the inputs arriving from the different senses such as when watching a movie, where the auditory and visual inputs are both likely to being attended to simultaneously, or when looking at the object that we happen to be palpating in our hands. The basic ability to process information from two or more sensory modalities simultaneously and to integrate that information in order to form coherent representations of the external world renders us multisensory creatures (e.g., Stein et al., 1996; Ernst and Bülthoff, 2004).

It can be argued that the interactions observed between vision and touch represent a special case of multisensory integration. For, unlike other combinations of the “spatial senses” (including the modalities of vision, touch, and audition), these two senses are very often stimulated at one and the same time. The reason for this being that tactile stimulation is almost always accompanied by some visual event, that is, by a potentially observable object touching the body surface. Hence, an organism can often use visual information in order to help predict impending tactile stimulation. Often, visual information can also be used to specify the location from which that stimulation happens to have originated in external space (see Gallace and Spence, 2014, for a review).

Due to its relevance to our everyday lives, the interplay between vision and touch has been investigated by a large body of research over the last 75 years or so (see Tastevin, 1937; Gibson, 1943 for early work), which has taken a variety of different approaches to the topic. While a number of researchers have utilized independent visual and tactile stimuli, other studies have investigated how vision of the body-part being stimulated can influence a participant’s performance in a purely tactile task. Strikingly, and irrespective of the approach that has been taken, many studies that have looked at interactions between the modalities of vision and touch can be classified as being, in some sense, spatial (cf. Spence, 2013). In many studies, this is because the participants have had to perform tasks that were explicitly spatial, such as, for example, in the orthogonal spatial-cuing paradigm, where the target property to be judged by the participant is its relative elevation (see Spence and Driver, 2004; Spence, 2013, for reviews).

Other studies that have utilized, for example, the recently repopularized rubber-hand illusion (RHI) paradigm (Botvinick and Cohen, 1998; see also Tastevin, 1937, for early work in this area), have tended to utilize a visuotactile illusion resulting from the misattribution of the location of one’s own limb in external space (see Makin et al., 2008, for a review; though see also Ehrsson et al., 2005). Meanwhile, many other studies have investigated the influence of variations in the direction of a participant’s gaze (and hence vision) either toward, or away from, the body-part that is being stimulated, on tactile perception. Overt visual (and hence spatial) attention is, by definition, associated with the current direction of a person’s gaze. As such, to the degree that visual attention may give rise to enhanced tactile information processing at attended locations, these studies were not designed to reveal visuotactile interactions outside the realm of spatial attention.

Since in one way or another the participants in these commonly utilized tasks have needed to attend to a specific location, it is unclear whether vision actually affects tactile information processing merely when/because gaze (i.e., overt spatial attention) happens to be directed toward the location in space where a tactile event subsequently happens to be presented. That is, when interpreting the results of such studies, spatial attention (or the misattribution of the location of one’s limb in external space) is a mechanism that can potentially explain the effects allegedly mirroring influences of vision on tactile information processing.

Recently, it has been suggested that vision and touch (as has been shown to be the case for other combinations of the spatial senses) likely interact in a “*what*” as well as in a “*where*” system (Spence, 2013; see, e.g., Schneider, 1969; Goodale and Milner, 1992; Creem and Proffitt, 2001, for the distinction of two pathways in the visual modality, and see, e.g., Reed et al., 2005; Van Boven et al., 2005, for this distinction in the tactile modality). Within such a dual-stream model, we are especially interested in the “what” system, that is, in the pathway by which vision influences the identification and identity- (rather than location-) based selection of tactile stimuli (see, e.g., Rock and Victor, 1964; Rock and Harris, 1967; Ernst and Bülthoff, 2004; Moeller and Frings, 2011, for a few of the studies that have, intentionally or otherwise, attempted to focus on visuotactile interactions within the “what” system).

In this review, the influence of vision on tactile information processing will be critically evaluated. In particular, we review the various evidence that supports a spatial, as well as a non-spatial, influence of vision on the processing of tactile distractors. In the first part of this review, however, we will consider the extant literature that has looked at the influence of vision on the processing of tactile targets. There, we present the results of spatial cuing studies and those studies that have investigated the impact of changes in the direction of a participant’s gaze on tactile information processing. Then, turning to those studies in which spatial influences have been controlled for, we go on to present evidence demonstrating that the speeded detection of tactile targets can be facilitated, and tactile resolution enhanced, at those locations on the body surface that can be seen (as compared to when vision of the body-part isn’t allowed; e.g., Tipper et al., 1998; Kennett et al., 2001b) even when the direction of a participant’s gaze is held constant.

To date, far less is known about the influence of vision on the processing of tactile distractors. Thus, in the second part of this review, we will take a closer look at the literature that has attempted to analyse the influences of distinct visual stimuli, gaze direction, and vision (or rather gaze direction) on tactile distractor processing. We will argue that vision appears to enhance the processing of tactile distractors by spatial as well as non-spatial means – just as is the case for tactile targets – even when vision is entirely irrelevant to a participant’s task.

## SPATIAL CONTRIBUTIONS TO THE INFLUENCE OF VISION ON THE PROCESSING OF TACTILE TARGETS

The research on visuotactile interactions that has been conducted to date can be broken down into two broad categories; on the one hand, both visual and tactile stimuli have been presented to test whether visual stimuli (e.g., cues or distractors) exert a significant influence over the processing of tactile events (e.g., targets) that happen to occur at around the same time. Here, tasks that are explicitly spatial have typically been used (Spence et al., 2004a). So, for example, in a number of studies, the location of the target has been the stimulus property that participants have had to respond to. In variants of the orthogonal spatial-cuing task (see Spence and Driver, 2004, for a review), as well as in variants of the crossmodal congruency task (see Spence et al., 2004b, 2008, for reviews), participants have often been required to discriminate whether a vibrotactile target presented to the thumb or index finger of either hand has been presented from one of the two upper locations versus from one of the two lower locations instead. Typically, participants have had to respond by making a speeded toe versus heel response to indicate the elevation of the target.

On the other hand, there are those studies in the literature in which the participants have either been instructed to direct their gaze toward the part of their body that is being touched, or to divert their gaze elsewhere. Within this group of studies, researchers have also compared participants’ performances in those conditions in which vision of the body-part that was being stimulated was available versus those conditions in which the participants have been blindfolded (and hence vision was unavailable). Another comparison that researchers have been fond of making is between those conditions in which the participants either have, or have not, been able to see the tactile stimulus impacting on their skin surface. Note that these studies implicitly inherit a spatial bias, since the participants had to direct their (visual and tactile) attention selectively toward a particular location rather than another. For each of these kinds of visual manipulation, we will outline the role of space, and thus highlight how it might contribute to the interaction of interest.

In order to specify the spatial constraints on any interactions between visual and tactile stimuli, many studies have implemented variants of the crossmodal spatial attentional-cuing paradigm (see Posner, 1978, 1980, for the original unimodal spatial-cuing paradigm of visual spatial attention). This has become a well-established tool used by researchers in order to investigate how attention is directed spatially by the presentation of a pre-cue (see Spence and Driver, 2004, for a review of the crossmodal cuing literature; see **Table 1**).

**Table 1:** Summary table highlighting that, irrespective of the approach taken, most published studies have provided evidence in support of the existence of visuotactile interactions.

Study	Task	Stimulus modalities	Gaze varied?	Vision of the body-part stimulated varied?	Modulation of touch by vision observed?
Chong and Mattingley (2000)	Exogenous spatial-cuing paradigm	V, T	No	No	na.
Kennett et al. (2001a)		V, T	No	No	na.
Kennett et al. (2002)		V, T	No	No	na.
Gray and Tan (2002)		V, T	No	No	na.
Spence et al. (2000a)		V, T	No	No	na.
Spence and McDonald (2004)		V, T	No	No	na.
Spence et al. (1998a)		V, T	No	No	na.
Spence et al. (2000b)		V, T	No	No	na.
Spence et al. (1998b)		V, T	No	No	na.
Spence et al. (2004b)		V, T	No	No	na.
Honoré et al. (1989)	Target-detection task	T	Yes	Yes	No
Tipper et al. (1998)		T	Yes	Yes	No
Tipper et al. (2001)		T	No	Yes	Yes
Driver and Grossenbacher (1996)	Target-discrimination task	T	Yes	Yes	No
Botvinick and Cohen (1998)	Rubber-hand paradigm	V (rubber hand), T	No	No	Yes
Gallace and Spence (2005)	Temporal order judgment task	V (mirror image), T	No	Yes	Yes
Guterstam et al. (2013)	Invisible-hand paradigm	T	No	No	Yes
Soto-Faraco et al. (2004)	Congruency task with mirror manipulation	V (mirror image), T	No	No	Yes
Kennett et al. (2001b)	Two-point threshold discrimination	V, T	No	Yes	Yes
Frings and Spence (2013)	Negative-priming paradigm	T	No	Yes	Yes
Wesslein et al. (in press)	Flanker paradigm	T	No	Yes	Yes

*Within this table the term *vision* describes whether a condition where vision of the body-part stimulated was provided is compared to a condition where vision of the body-part stimulated was prevented irrespective of whether vision was manipulated by blindfolding the participants or by selectively occluding the body-part stimulated. V = visual stimuli were presented; T = tactile stimuli were presented.*

In a typical exogenous study, spatially non-predictive visual pre-cues are presented shortly before tactile targets (or vice versa). Importantly, in the unimodal as well as the crossmodal variants of this task, the participant has to judge the external location (normally the elevation) from which the target stimulus has been presented (e.g., Kennett et al., 2001a, 2002; Spence and McGlone, 2001), and thus their task is inherently spatial in nature (see Spence and McDonald, 2004; Spence et al., 2004a, on this point).

It is now well-known from those visuotactile studies that have used the crossmodal orthogonal spatial-cuing paradigm^1^ (and where the cue does not elicit a response bias; see e.g., Spence and Driver, 1997) that the responses of participants toward those tactile targets that happen to be presented from the same location (or side) as the visual pre-cues tend to be faster and more accurate than their reactions toward the same targets when the visual cue happens to be presented from the other side of central fixation instead (e.g., Kennett et al., 2002). Such a pattern of performance facilitation has normally been explained in terms of an exogenous shift of spatial attention. As an aside, if the temporal interval between the onset of the visual cue and the tactile target is increased, then the facilitation that is normally observed at the cued location can sometimes be replaced by a longer-lasting inhibitory aftereffect, known as inhibition of return (IOR; e.g., Spence et al., 2000a).

**FIGURE 1:**
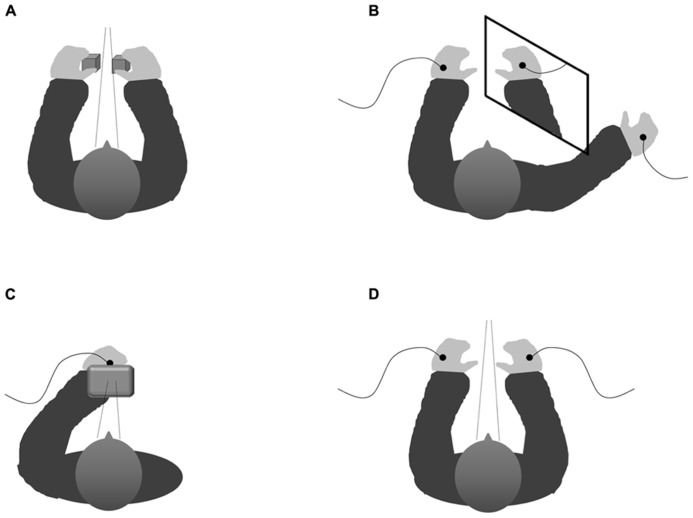
**Schematic configuration showing the various experimental set-ups implemented within the studies that have analyzed the influence of vision on tactile targets.** **(A)** Experimental set-up of the typical visuotactile congruency task (cf. Spence et al., 2004c). **(B)** Experimental set-up in which a mirror is used to elicit the perceptual illusion of both hands being positioned close together though they are actually positioned far from each other (Soto-Faraco et al., 2004) thus causing interference between the visually and the proprioceptively defined locations of the participant’s own right hand in a way that allows one to examine whether vision or proprioception determine the allocation of spatially selective attention. **(C)** Experimental set-up implemented within our response-priming paradigm (with the right hand placed behind a screen; cf. Mast et al., unpublished manuscript) which represents a non-spatial tactile selection task where the direction of gaze is controlled for by presenting visual and tactile cues from roughly the same location. **(D)** Schematical depiction of the experimental set-up implemented within our tactile flanker paradigm (Wesslein et al., in press) notwithstanding the spatial dimension by keeping gaze direction constant by using a chin rest as well as presenting on fixation cross on the computer screen situated centrally in front of the participant on each trial. See text for details.

Those studies that have used the crossmodal congruency task (see **Figure 1A**, for a schematic figure of the experimental set-up) have typically demonstrated that a participant’s performance in a speeded elevation-discrimination task is impaired when visual distractors are presented from an incongruent elevation with respect to the tactile target than when both target and distractor happen to be presented from the same (i.e., congruent) elevation (see Spence et al., 2004c, 2008, for reviews). The “crossmodal congruency effect” is largest when the stimuli are presented from the same lateral position (or side of fixation), and has been shown to fall off as the lateral separation between the target and distractor increases (e.g., as when the target and distractor are presented to separate cerebral hemispheres).

The results of the large number of studies that have been conducted over the last decade or so using either one of these experimental paradigms – the crossmodal orthogonal spatial-cuing paradigm or the crossmodal congruency task – have generally converged on the conclusion that the relative location from which the multisensory stimuli have been presented determines the degree to which they exert an influence over one another (excepting any effects that can be attributed to mere eccentricity effects).

Referring to the distinction between *exogenous* and *endogenous* spatial cuing, there is now robust evidence to support the role of space in both types of crossmodal spatial-cuing paradigms (e.g., Spence et al., 2000b; Driver and Spence, 2004). What this means is that the relative location of visual and tactile stimuli determines visuotactile interactions in both a “bottom-up” as well as a “top-down” manner. More specifically, in those studies that have used the exogenous spatial-cuing paradigm, the influence of a salient pre-cue on a participant’s reaction toward a subsequently presented target has been investigated. As a result, the pre-cue is non-informative with regard to the likely location (or identity) of the target (and may thus be regarded as a to-be-ignored task-irrelevant distractor). Consequently, the target is as likely to occur in the same location as the pre-cue as it is to occur at a different one; thus, the *stimulus-driven *effect of a pre-cue on a target is obtained within exogenous spatial-cuing paradigms (e.g., Spence et al., 1998a, 2000a; Chong and Mattingley, 2000; Kennett et al., 2001a; Gray and Tan, 2002; Spence and McDonald, 2004; see also Spence et al., 2004a, for an overview of the crossmodal research utilizing the exogenous spatial-cuing paradigm).

By contrast, in those studies that have attempted to investigate endogenous spatial-attentional cuing, a pre-cue that is predictive with regard to the location of the target has been documented to give rise to attentional shifts. Thus, within the endogenous cuing paradigm, the *top-down* crossmodal effects of a pre-cue on a target have been examined (Spence et al., 2000b; see also Spence et al., 1998b, 2004b; Driver and Spence, 2004, for a review).

Importantly, visuotactile interactions have largely been obtained within both variants of the crossmodal spatial-cuing paradigm, despite the striking differences that have sometimes been observed between exogenous and endogenous spatial attention (see Spence and Driver, 1994; Klein and Shore, 2000; see also Spence and Gallace, 2007, for exogenous and endogenous attentional effects specifically in the tactile modality), thus indicating that space (supramodally) moderates stimulus-driven as well as top-down effects between vision and touch.

The available research that has been published to date therefore suggests that it is the *relative location* from which the visual and tactile stimuli are presented in external space that determines the magnitude of any crossmodal spatial-cuing effects. So, for example, holding the hands in a crossed posture causes a reversal of the observed effects in exogenous (Kennett et al., 2002) as well as in endogenous cuing paradigms (Spence et al., 1998b, 2004b): a visual cue presented on the left (right) side elicits more pronounced interference effects for tactile targets presented on the right (left) hand when the hands are crossed. The same crossing effect has also been documented in those studies that have used the crossmodal congruency task (see Spence et al., 2004c, 2008, for reviews). Hence, irrespective of the posture adopted by the participant’s hands, the influence of vision on tactile information processing is especially pronounced when the visual distractor occurs on the same side of external space as the tactile target. This result means that it is the location of the stimuli in external space, rather than their initial hemispheric projections, that is the crucial factor when it comes to determining how space moderates the integration of visual and tactile stimuli (see Sambo and Forster, 2009, for supporting evidence from an event-related potentials, ERP, study), at least in neurologically normal participants (see Spence et al., 2001a,b, for patient data; see also Valenza et al., 2004, for the effects of changes to the posture of the hands on the tactile discrimination performance in a patient with bilateral parietal damage).

The influence of vision on tactile information processing has been analyzed using the *attentional blink* (AB) paradigm. The AB refers to an impairment in responding to a target that happens to be presented after another target that requires a response, as compared to a target that happens to be presented after another target that does not require a response. Besides the well-established AB that has been documented repeatedly in the visual modality over the last couple of decades or so (see Raymond et al., 1992, for the original study), an AB has also been demonstrated in both the auditory (e.g., Soto-Faraco and Spence, 2002) and tactile modalities as well (Hillstrom et al., 2002; Dell’Acqua et al., 2006). Importantly, however, with regard to the scope of the present review, Soto-Faraco et al. (2002) reported evidence in support of the existence of a crossmodal visuotactile AB. Given that Soto-Faraco et al. (2002). implemented a spatial-localization task (i.e., a speeded target elevation-discrimination task), this result is in line with the evidence obtained within crossmodal spatial-cuing paradigms in highlighting that visuotactile interactions may be more apparent in those tasks where space is somehow relevant to the participant’s task (cf. Spence, 2013). As an aside, note that the asymmetrical pattern of results in the blocked conditions indicates that responses associated with visual stimuli exhibited a stronger aftereffect over subsequent target processing than responses associated with tactile stimuli.

Building on the research demonstrating that a neutral visual stimulus enhances the processing of co-located tactile stimuli that happen to be presented subsequently, Poliakoff et al. (2007) demonstrated the modulation of the magnitude of this visuotactile spatial-cuing effect by the threat value of the visual stimulus (i.e., threatening pictures of snakes and spiders vs. non-threatening pictures of flowers and mushrooms). That is, threatening visual cues enhanced tactile processing at the pre-cued location more than did non-threatening visual pre-cues, indicating that threat value modulates the amount of (spatial) attention allocated to a visual stimulus, thereby influencing the processing of a subsequent tactile target at exact this location.

From a somewhat different viewpoint, Poliakoff et al.’s (2007) results indicate that proximity to the hands can increase the amount of attention that is allocated to a threatening stimulus. As an aside, then, Abrams et al. (2008) demonstrated that proximity of the hands can also augment the capture of attention by a non-threatening visual stimulus. In their study, proximity of the hands (hands held close to vs. far from the display where visual stimuli happened to be presented) moderated visual search, visual IOR, and visual AB. That is, the processing of visual stimuli was prolonged for those stimuli near the hands (i.e., participants were slower to disengage their attention from those visual stimuli close to the hands) as compared to those far from the hands. These results show that the disengagement of attention from visual stimuli is delayed near the hands. Thus, proximity to the hands can be concluded to alter visual information processing.

Yet, going beyond the investigation of effects of proximity to the hands (and again investigating tactile information processing), Van Damme et al. (2009) implemented a similar experimental set-up as Poliakoff et al. (2007) but compared the effects of visual stimuli showing different types of threat, namely the threat of physical harm to the hand (which was the body region receiving the tactile stimulation in this study) versus general threat to the whole person. Extending Poliakoff et al.’s (2007) findings, these researchers demonstrated that physical threat *selectively* elicited a shift of their participants’ tactile spatial attention. This was reflected in the prioritization of tactile information presented at the hand positioned at about the same location where the visual pre-cue showing physical threat had happened to occur over tactile information presented to the other hand. By contrast, the shift of auditory spatial attention was not modulated by the type of threat. Hence, auditory spatial attention may generally be enhanced in threatening situations, while the amount of attention captured by a tactile stimulus delivered to the hands further depends on the degree of apparent threat of physical harm toward specifically this body-part. Summing up, it seems that not only does the proximity of the hands to (threatening) visual stimuli determine the allocation of spatial attention but also can the focus of tactile spatial attention precisely be guided by the information which body-part it is that is threatened.

What we have covered so far in this review are those tasks concerned with *covert* spatial attention within the visual modality. In these tasks, participants focus their visual attention on a specific location without making any overt head, eye, or bodily movements (e.g., Spence et al., 1998a, 2000b; Kennett et al., 2002). Importantly though, the presentation of a visual stimulus has also been demonstrated to enable faster and more accurate saccades toward a to-be-detected tactile target (i.e., speeded *overt*-orienting response) if the visual stimulus is located at approximately the same spatial position (Diederich et al., 2003). Note here that covert and overt tactile spatial attention are typically linked, but – as for vision – can be separated under a subset of experimental conditions (e.g., Rorden et al., 2002). In conclusion, it seems that the magnitude of visuotactile interaction effects elicit by covert as well as overt spatial orienting is moderated by the distance between the visual and tactile stimuli involved.

In line with the assumption of a spatially specific influence of vision on touch, Valenza et al. (2004) reported a facilitation of tactile discrimination performance on trials where a visual distractor was presented on the same side as the tactile target as compared to trials where the visual distractor was presented on the opposite side. What’s more though, is that this facilitation was observed only in healthy individuals but not in a patient with bilateral parietal damage. Still, the patient’s left-hand responses were speeded up by a concurrent visual distractor (as compared to when no visual distractor was presented) irrespective of whether the distractor occurred at the right or the left side. As these are results from a single-case study, they provide only indicative evidence. Still, it should be noted that these results suggest that there are *spatial* mechanisms by which visual stimuli can affect tactile information processing but that, in addition, vision also exerts a spatially non-specific influence over tactile information processing.

Rather than using distinct visual and tactile stimuli, several researchers varied whether or not their participants were able to see the body-part receiving the tactile stimulation by manipulating the direction of participants’ gaze. For example, faster tactile target detection has been reported when the eyes are directed toward the stimulated region on the skin surface than when they are directed toward another area (in the same or the opposite visual hemisphere; Honoré et al., 1989).

Accordingly, comparing two conditions within the same group of participants whose hands were occluded from view by cardboard boxes, namely a condition where the gaze was directed toward the hand receiving the tactile stimulation to a condition where the gaze was directed toward the other hand, Tipper et al. (1998) demonstrated tactile target detection to be faster in the former condition. Remarkably, these studies imply influences of the visual modality on the performance of a purely *tactile* task.

Still, the observed effects of gaze direction might reflect effects of spatial attention toward the body-part that is being stimulated. More specifically, the direction of gaze toward the body-part stimulated might enhance processing of stimuli occurring within the respective region on the body surface, thus causing the effects reported. In the studies of tactile target detection that have just been reviewed, the contributions of vision and gaze direction to tactile perception cannot be disentangled from these effects attributable to attention.

Combining the approaches of presenting a visual pre-cue prior to tactile stimulation and of manipulating whether vision of the body-part stimulated is provided, it has been demonstrated that visual events increase the probability of participants erroneously reporting a tactile sensation (as measured by the Somatic Signal Detection Task). However, this only holds true when vision of the stimulated body-part (i.e., the hand) is provided (Mirams et al., 2010). This finding emphasizes that non-informative visual stimuli may not only enhance the processing of tactile stimuli occurring at roughly the same location, but may also interfere with tactile processing, thus possibly leading to the sensation of touch in the absence of any actual stimulation. In other words, the direction of spatial attention toward a body-part that the participant expects to receive a tactile stimulus can have detrimental effects on tactile target detection (i.e., it can give rise to higher false-alarm rates).

Taken together, the findings presented so far show that what a person sees can affect their tactile perception and facilitate responding to a tactile target. Still, it remains an open issue the degree to which the influences that have been obtained result from spatial attention processes elicited by gaze direction, and whether vision can influence tactile target processing in a non-spatial fashion.

## HOW VISION INFLUENCES TACTILE TARGET PROCESSING EVEN WHEN SPACE IS COMPLETELY TASK-IRRELEVANT

In this section, we review those studies that have investigated whether tactile information processing can be influenced by vision while the direction of gaze (and thus the direction of spatial attention) is held constant. Strikingly, these studies still provide evidence in favor of the influence of vision on the tactile modality, thus suggesting mechanisms beyond those mentioned so far that underlie the influence of vision on touch.

In order to provide insights regarding the influence of vision on touch *albeit* the effect of the spatial domain, the experimental procedures implemented need to meet some important criteria. Most importantly (and unlike the crossmodal spatial-cuing tasks presented in the previous section), those tasks in which the to-be-judged target property is not spatial have to be used. Furthermore, the direction of gaze needs to be controlled for, since it represents a spatial confound when the influence of vision of a body-part being stimulated is under investigation.

Going beyond the influence of gaze direction, Kennett et al. (2001b) have reported that vision of the body-part stimulated *per se* can enhance tactile resolution. Control of the direction of gaze and thus of spatial attention was achieved by comparing two conditions with the participant’s gaze being directed toward the same location in both conditions. While in one condition the participants were able to see the body-part that was being stimulated (i.e., the forearm) shortly before the stimulus was delivered, participants in the other condition were presented with a neutral object that appeared as though it was positioned at the location to which the stimulation was being delivered.

Tactile resolution, as assessed by means of the two-point discrimination threshold, was enhanced when vision of the body-part that was about to receive a tactile stimulus was provided (as compared to when gaze was directed to the same location but the body-part was occluded by a neutral object). Importantly, vision at the moment when the tactile stimulus touched the skin surface was prevented in any case. Hence, the observed results indicate that vision (beyond the orienting of gaze) can enhance the sensitivity of the tactile receptor field corresponding to the visually attended region on the body surface (see also Haggard et al., 2007; Cardini et al., 2011). Similarly, performance in a tactile orientation-discrimination task was enhanced when the body-part stimulated (the hand in this case) rather than a neutral object was viewed, even if the neutral object in the latter condition was seen at the location of the body-part that was stimulated (see Cardini et al., 2012). Finally, seeing a hand has been shown to enhance tactile acuity on the face (Serino et al., 2009).

Utilizing a different approach to control for the influence of gaze direction, Tipper et al. (1998) provided one group of participants with *indirect* vision of one of their hands via a real-time image of their right versus left hand on a video monitor placed at the body midline. In particular, their gaze was never directed toward the real hand in this condition. In contrast, a second group of participants oriented their head and eyes toward their right versus left hand, and was thus provided with *direct* vision. In both conditions, tactile target detection was faster for those targets occurring on the hand viewed as opposed to the other hand, implying that vision without gaze affects tactile detection. What remains unclear, however, is whether directing attention to the body-part stimulated in another way, as, for example, by presenting participants with the word describing it, would also be sufficient to induce enhanced tactile information processing.

Nevertheless, the findings obtained so far at least provide suggestive evidence that vision may enhance the sensitivity of the tactile receptors on those locations on the body surface that are visually attended. Notably, this assumption is further supported by the results of yet another study in which tactile spatial detail has been demonstrated to be even further enhanced when the participant’s view of the body-part that had been stimulated is magnified (i.e., when viewing the arm through a magnifying glass) than when seeing it without magnification (Kennett et al., 2001b). Importantly, however, in neither condition of this study could the arm be seen at the moment when the tactile stimulus impacted on the participant’s skin.

One could argue that some kind of “habitual effects” underlie the effects of the studies by Tipper et al. (1998) and Kennett et al. (2001b) as participants are used to seeing the body-parts that were stimulated in these studies (i.e., the hand and the forearm). Yet, overcoming this limitation, Tipper et al. (2001) replicated Tipper et al.’s (1998) earlier findings using a body-part that is usually unavailable for proprioceptive orienting (namely the back of the neck). In sum, the results of these studies indicate that vision generally enhances the speed of tactile target detection and tactile resolution at the visually attended location on the body surface.

Here, mention should also be made of the studies conducted by Graziano and Gross (1992, 1993; see also Desimone and Gross, 1979; Bruce et al., 1981; Hikosaka et al., 1988), which revealed that there are bimodal visuotactile neurons in macaque monkeys (e.g., in the face- and the arm-region of the somatotopically organized putamen). For these neurons, the tactile receptive field has been demonstrated to approximately match the visual receptive field, meaning that these neurons respond to visual and tactile stimuli at the same location on the body surface. When the arm is moved, the visual receptive field thus moves with it (Graziano and Gross, 1994). The finding that visual information about a specific body-part enhances tactile detection performance as well as tactile resolution on this specific body-part may be attributable to such neurons responding to visual and tactile stimuli at the same location on the body surface (see Graziano et al., 2004, for an overview of multimodal areas in the primate brain).

Attempting to analyse another potential pathway by which vision might affect tactile information processing, we developed a visuotactile response-priming paradigm in order to investigate whether visual stimuli hamper the processing of tactile stimuli if they are associated with distracting information. More specifically, we addressed the question of whether responses that are associated with irrelevant visual pre-cues interfere with (or facilitate) the responses that are elicited by tactile targets that happen to be presented at about the same time and vice versa (Mast et al., unpublished manuscript). To control for the effects of variations in spatial attention, the visual and tactile stimuli were presented from roughly the same location in external space. Therefore, participants positioned the hand to which the tactile stimuli were to be delivered directly behind a small monitor on which the visual stimuli were presented (see **Figure 1C**). Note that given this experimental set-up, the participant’s spatial attention was always directed toward the same position irrespective of the mapping of the pre-cues and targets to modalities.

Within the response-priming paradigm developed by Mast et al. (unpublished manuscript), all of the stimuli – both the pre-cues and targets – were associated with one of two responses. Hence, on each trial, the pre-cue and the target could be mapped onto the same responses (these are known as compatible trials) or opposite responses (known as incompatible trials). The participants were instructed to ignore the pre-cue and to discriminate which of the two possible targets had been presented according to the target-intensity (for the visual modality, the targets differed with regard to their brightness; for the tactile modality, these differed with regard to their amplitude). Thus, there were four different stimuli: one high intensity visual stimulus, one low intensity visual stimulus, one high intensity tactile stimulus, and one low intensity tactile stimulus.

The presentation of the visual pre-cues exerted a significant crossmodal influence over tactile target processing, that is, response latencies were significantly shorter in the compatible trials than in the incompatible trials. In other words, a significant response-priming effect was observed. This result shows that vision can aid tactile information processing by facilitating the retrieval of relevant information (here the S-R mapping) from memory, as, for example, by pre-activating the to-be-executed response.

Remarkably, no significant response-priming effect emerged when tactile pre-cues preceded the visual targets. Note that these contrary results as a function of the mapping of pre-cues/targets to modalities cannot be attributed to the operation of spatial attention (since spatial attention would have been expected to lead to comparable response-priming effects in both directions). Rather, these results suggest that the information that is attached to visual stimuli (associated responses in this case) is either more automatically retrieved from memory than the information that is associated with tactile stimuli or else that it is more difficult to inhibit those responses that happen to be elicited by task-irrelevant *visual* pre-cues than to inhibit those responses that are elicited by task-irrelevant *tactile* pre-cues. Both possible mechanisms may contribute to the stronger response-priming effects from vision to touch than in the opposite direction. As an aside, in Soto-Faraco et al.’s (2002) study, an asymmetrical visuotactile AB has accordingly been obtained. With experimental blocks in which the visual target constantly led the tactile target or vice versa, these researchers reported a crossmodal AB only in the former condition (see also Dell’Acqua et al., 2001, Experiments 3–4). This is further evidence pointing to the conclusion that the information associated with visual stimuli somehow dominates over information attached to tactile stimuli.

Note that these mechanisms may also play a role within the crossmodal congruency paradigm. First, the *response-competition* account explaining the crossmodal congruency effect also inherits the idea that pre-cues elicit the retrieval of a particular response (or response tendency) even if no response to the stimulus is required. In the case of distractors presented from a location that happens to be different from the subsequent target location, this tendency is incongruent with the required response, whereas in the case of distractors presented from the same location as the subsequent target, it is congruent with the required response. Consequently, a response conflict is only present in the former condition, possibly contributing to the observed visuotactile effect (see Shore et al., 2006). Second, corroborating the pattern of results obtained within our response-priming paradigm, crossmodal congruency effects from vision to touch have been found to be stronger than those from touch on vision (Spence et al., 2004c; Walton and Spence, 2004; Spence and Walton, 2005). These findings are further in line with the body of evidence indicating a generalized bias of attention allocation toward the visual modality (e.g., Posner et al., 1976; Spence et al., 2001c).

Summing up, in those studies that have controlled for the influence of the spatial dimension, an influence of vision on tactile target processing is still observed. The evidence suggests, on the one hand, that vision enhances the processing of tactile stimuli applied to tactile receptor fields that correspond to the viewed locations on the body surface and, on the other, that visual stimuli can prime categorization responses to tactile targets when gaze is kept constant.

## HOW SPACE CONTRIBUTES TO THE INFLUENCE OF VISION OVER TACTILE DISTRACTOR PROCESSING

Most studies that have examined the influence of vision on tactile information processing have been concerned with the processing of tactile targets; that is, researchers have typically analyzed whether vision modulates responses to tactile *targets*. Consequently, much less is known about the influence of vision on tactile *distractor* processing, that is, on tactile stimuli that should be ignored or are irrelevant for (or may even interfere with) responding. One exception is a series of experiments that were conducted by Driver and Grossenbacher (1996). These researchers presented results suggesting that vision, guided by the direction of gaze, not only exerts an influence over tactile target processing but also over tactile distractor processing. In their study, a tactile target and a tactile distractor were delivered to the participant’s right and left little fingers, respectively. Driver and Grossenbacher (1996) separately analyzed the influences of both vision (i.e., participants were blindfolded vs. not blindfolded) and gaze direction on performance.

More effective tactile selection (i.e., lower differences in the latencies on those trials with distractors dissimilar to the targets as compared to trials with distractors similar to the targets) was observed when the participant’s gaze was directed toward the finger that received the target than when their gaze was directed toward the finger receiving the distractor. Note, once again, that this finding implies that tactile information processing is generally enhanced at those locations where gaze happens to be directed, irrespective of whether the tactile stimuli happen to be targets (and therefore relevant with regard to the task at hand) or distractors (and therefore irrelevant with regard to the task at hand).

Accordingly, even in blindfolded participants, Driver and Grossenbacher (1996) observed less effective (or efficient) tactile selection when the hands were placed close together in external space than when they were placed far apart. This result is in line with the assumption that gaze direction generally enhances tactile information processing, as both the target and the distractor might have been positioned within the direction of gaze when the distance between the target and the distractor location was small. Thus, given the small distance between the participant’s hands, spatial attention (as elicited by the direction of gaze) is likely to be simultaneously directed toward both the target and the distractor location. As a result, the processing of both the target and the distractor should be enhanced in the hands-close condition but not in the hands-far conditions (where the gaze, and therefore spatial attention, are selectively directed toward either the target or the distractor location), in turn, causing a stronger interference from dissimilar as compared to similar distractors within the former condition.

Somewhat differently, Soto-Faraco et al. (2004) gained strong support for the influence of vision over tactile information processing by demonstrating that the visually perceived distance between a participant’s hands affects tactile selection when it is at odds with the actual proprioceptively specified distance. Therefore again simultaneously receiving a vibrotactile target and distractor stimulation on the previously defined target and distractor hand, respectively, their participants had to perform a speeded target elevation-discrimination task. In the critical experimental condition, a mirror was positioned vertically close to the participant’s right hand, in a way that the participants had the visual impression of their left hand lying close to their right hand (although they could actually see a mirror-image of their right hand with their left hand being placed further apart from the right hand than the mirror-image; see **Figure 1B**). Just as in a hands-close condition without the mirror, tactile selection was less effective (i.e., the detrimental impact of a dissimilar as compared to a similar distractor was more pronounced) in this mirror-condition than in the hands-far condition without the mirror.

In another study in which a mirror was used to vary the visually perceived distance between the hands, participants performed a temporal order judgment (TOJ) task with tactile stimuli being presented to either index finger (Gallace and Spence, 2005). Significant performance differences were observed as a function of the participant’s perceived hand separation (elicited by means of the mirror reflection of the own left hand). Performance was significantly worse when the participant’s hands appeared visually to be close together than when the hands appeared at either middle or far distances. Importantly, just as was the case in the study by Soto-Faraco et al. (2004), the observed pattern of results was consistent with that obtained when the proprioceptively specified distance between the hands had been varied (investigated in a dark room, where vision of the hands was prevented; see Shore et al., 2005).

Although the results of these studies varying the visually perceived separation between the hands cannot be explained in terms of the direction of spatial attention by variations of the orientation of gaze, they are nonetheless highly dependent on space. Indeed, they point to a further mechanism by which space may contribute to the influence of vision on tactile information processing. More specifically, as the participant’s hands are falsely perceived to be positioned near one another in the mirror-condition, these results indicate that visual information exerts a more profound effect on the spatial distribution of tactile selective attention than proprioceptive information concerning the distance between the hands. Consequently, the illusory visual perception of the left hand being positioned close to the right hand may lead to the allocation of attention onto the hand hidden behind the mirror as if that hand were actually positioned at the visually defined location.

Taken together, then, the findings presented in this section of the review demonstrate, on the one hand, that the direction of gaze toward the stimulated body-part enhances the processing of to-be-ignored tactile stimuli (i.e., distractors) just as it enhances the processing of tactile target stimuli independently of vision, possibly by guiding a participant’s spatial attention. On the other hand, they show that the interference between tactile target and distractor stimuli crucially depends on the visually perceived relative location of tactile target and distractor stimuli rather than on their proprioceptively specified relative location.

## HOW VISION INFLUENCES TACTILE TARGET PROCESSING EVEN WHEN SPACE IS TASK-IRRELEVANT

When controlling for the direction of gaze and thereby usually for spatial attention (although one could of course always argue that it is possible that covert attention and gaze are directed toward different locations in external space), tactile selection tasks represent an especially useful tool with which to examine non-spatial influences of vision on tactile distractor processing. This is because, in these experimental studies, the effects of spatial attention as well as any attentional effects elicited (explicitly or implicitly) by the nature of the task instructions (namely to attend to the location where the tactile target will occur rather than to the distractor location) are controlled for. Note that Driver and Grossenbacher (1996) also used a tactile selection task in order to examine the influence of vision on target *and* distractor processing. However, to the extent that these researchers investigated the effects of the direction of gaze at the same time as they assessed the effects of vision, their results might be attributable to the variation of the direction of spatial attention by gaze.

Furthermore, Driver and Grossenbacher (1996) did not obtain any *crossmodal* effect of vision on tactile selection (i.e., no differences in performance were observed between blindfolded and sighted participants). It is, however, important to note that any potential effects here may have been masked by the effects of spatial attention. In this sense, our own more recent research can be seen as complementing Driver and Grossenbacher’s earlier findings. More specifically, we utilized a negative-priming paradigm and a flanker paradigm in order to investigate how vision influences the processing of tactile distractors.

Implementing a tactile variant of the negative-priming paradigm, Frings and Spence (2013) conducted a study designed to compare a condition in which the participant’s hands were positioned close together/touching with a condition in which their hands were positioned far apart. In both cases, the participants were unable to see their limbs since they were occluded from view by a cover (see their Experiments 2 and 3). The participants were presented with two vibrotactile stimuli at a time, one delivered to either hand. They were instructed to ignore one of these stimuli while responding to the other vibration as rapidly and accurately as possible (a color cue was presented on the screen to indicate whether the participants should respond to the vibrotactile stimulus presented to their right hand or the stimulus presented to their left hand).

Tactile negative-priming effects were computed as the slowing of response latencies in those (probe) trials in which the target constituted the vibrotactile stimulus that had been presented as the distractor (and thus had to be ignored) in the preceding trial (i.e., the prime trial), as compared to response latencies in those probe trials in which the vibrotactile targets had not been presented in the prime trial. Overall, the data revealed that the influence of the distance between the hands was qualified by a disordinal interaction with vision. This means that, while significant negative-priming effects were obtained when the participants’ hands were occluded from view in the hands-close condition, they disappeared when the participant’s hands were visible in this posture. The presence of a disordinal interaction implies that significant negative-priming effects were also obtained when the participants’ hands were visible in the hands-far condition but not when the hands were occluded from view.

Note here that in Frings and Spence’s (2013) study, the attention of the participants should have been directed to the target hand while performing the tactile selection task. Hence, the observed influence of vision on tactile information processing likely represents an effect that occurs regardless of a participant’s voluntarily guided (spatial) attention. However, this study did not provide any information concerning the mechanism by which vision influences the processing of tactile distractors. In this regard, the Eriksen flanker paradigm (see Eriksen and Eriksen, 1974, for the original study conducted within the visual modality; and Chan et al., 2005, for its extension to the auditory modality; see also e.g., Evans and Craig, 1992; Craig, 1995; Craig and Evans, 1995, for tactile variants of the paradigm) provides a useful tool with which to investigate the depth of distractor information processing.

As in the negative-priming paradigm, a target and a distractor are presented simultaneously with each of the four stimuli possibly serving as target or as a distractor. Consequently, another common feature is that not only are the targets associated with a response but so too are the distractors. The crucial aspect of the flanker-interference paradigm, however, is that a 4-to-2 mapping is used, meaning that the four stimuli are mapped onto two responses. As a result, three types of trials can be distinguished along two dimensions, namely the dimension of perceptual congruency, whereby trials with distractors that are identical to the current target are compared to those trials on which the distractors are different (i.e., perceptually incongruent) from the target, and the dimension of response compatibility, whereby trials with distractors that are mapped onto the same response as the current target are compared to those trials in which the distractors are mapped onto the opposite response.

Two different interference effects can be computed reflecting these dimensions, the so-called flanker-interference effect at the level of perceptual congruency (calculated by comparing perceptually congruent with perceptually incongruent trials), and the so-called flanker-interference effect at the level of response compatibility (by comparing response-compatible with response-incompatible trials). The occurrence of flanker effects allows one to draw conclusions as to the level to which the distractors have been processed: if there is interference only at the level of perceptual congruency, then it implies that the distractor stimulus was not processed up to the level of response preparation. By contrast, if the distractor is processed up to the level of response preparation, then the responses elicited by the target and the distractor would be expected to interfere in response-incompatible trials (but not in the response-compatible trials), resulting in a flanker effect at the response level.

Note that those studies investigating tactile congruency effects (e.g., Driver and Grossenbacher, 1996; Soto-Faraco et al., 2004; Gallace et al., 2008; Frings and Spence, 2010) have typically implemented a paradigm inspired by the Eriksen flanker paradigm. Yet, strikingly, only incongruent and congruent trials have been compared and hence it has not been possible to separate the effects of perceptual and response compatibility.

To investigate the crossmodal influence of vision on the depth of tactile distractor processing, we implemented a tactile variant of the 4-to-2 Eriksen flanker paradigm (see also Evans and Craig, 1992; Craig, 1995; Craig and Evans, 1995). Participants simultaneously received two tactile stimuli every trial (see **Figure 1D**, for the experimental set-up). Once again, one of these stimuli was presented to either hand, with the blockwise instructions to attend to the stimuli presented onto one hand (i.e., the target hand), while ignoring the distractor stimuli presented to the other (i.e., distractor) hand. In order to control for any influence of (overt) spatial attention, we kept the direction of gaze constant. Furthermore, the participant’s hands were placed next to each other, separated by a distance of about 40 cm, which makes it unlikely that spatial attention covers the external space including both hands, since participants appear to be able to split their attention between the two hands (Craig, 1985, Experiments 4–5; see also Craig, 1989). Next, we compared a condition in which the participants were blindfolded to another condition in which the participants were provided with a complete view of the experimental set-up (Wesslein et al., in press). Interestingly, vision was found to enhance the processing of tactile distractors from the perceptual level all the way up to the level of response preparation: while flanker effects at both levels were observed in the full-sight condition, only the perceptual flanker effect was apparent in the blindfolded condition.

The differential effects reported in the conditions with blindfolded and seeing participants cannot be accounted for in terms of the effects of spatial attention, since that should have been directed toward the target hand in both conditions. Hence, spatial attention need not be directed toward the location at which a tactile distractor is delivered in order for vision to influence its processing. Furthermore, the crucial effect of vision was concerned with irrelevant tactile stimuli suggesting that attention need neither be voluntarily guided toward the location at which a tactile stimulus happens to occur for vision to exert an influence over tactile information processing. Importantly, then, the pattern of results provides some of the first evidence to suggest that vision alone may give rise to a deeper processing of both tactile target *and* distractor stimuli (namely to their processing up to the response level), thus supporting the view that there can be a strong crossmodal influence of vision on tactile information processing through a process of enhanced tactile processing by vision of the (non-attended) body-part stimulated.

Taken together then, these results suggest that vision affects tactile distractor processing beyond its role in guiding a participant’s spatial attention toward the location of the tactile distractor. In fact, we have found evidence to demonstrate that vision might influence how deeply a tactile distractor is processed (e.g., whether it is processed up to the level of response selection) or how the eccentricity between tactile targets and distractors, that is, their distance from the body midline or maybe also the separation between them, is perceived.

## SUMMARY AND CONCLUSION

We have outlined the various ways in which vision influences the processing of tactile targets as well as tactile distractors. Discussing the cognitive mechanisms that may underpin such effects, we have attempted to highlight the important role that space plays in many of the crossmodal studies that have been published to date. Consequently, the visual modality – that is, either the presentation of distinct visual stimuli, the direction of gaze, and the visually perceived location of one’s limbs in external space – was suggested to affect the allocation of spatial attention relative to the body-parts, thus enhancing the processing of tactile stimuli at visually attended locations. What’s more, the information that was associated with irrelevant visual stimuli was demonstrated to interfere with information associated with tactile stimuli. The information associated with visual stimuli has thus been suggested to be automatically retrieved from memory, thus impairing tactile performance. As such, we have also presented a number of findings that together point to there being an influence of vision on touch that is independent of the spatial dimension (see **Table 1**). In reviewing the latter studies, we have highlighted how vision albeit the orientation of gaze affects the processing of both tactile target and distractor stimuli, for example, by furthering the sensitivity of the tactile receptor fields seen.

At present, knowledge concerning the influence of vision on tactile distractor processing is relatively scarce. Yet, one may ask whether there is any need to discuss the influence of vision on tactile targets and tactile distractors separately. Here, it is important to note that tactile targets will likely always receive attention since the participant has to respond to them in one way or another. By contrast, tactile distractors have to be ignored and would, presumably, ideally not receive any attention. As a consequence, one might argue that vision can have different influences on the processing of to-be-attended and to-be-unattended tactile stimuli: so, for example, one could argue that vision of the location where a (previously) unattended tactile stimulus happens to occur might have a larger impact on tactile information processing than vision of the location where an attended stimulus happens to be delivered (as the latter will receive attention in any way). However, concerning the impact of the guidance of spatial attention due to vision or gaze on tactile information processing, it can be concluded that there is no difference between the processing of tactile targets and tactile distractors. In particular, while responding to tactile targets is typically facilitated due to visually guided spatial attention (e.g., Honoré et al., 1989), interference from tactile distractors is increased due to visually guided spatial attention (Driver and Grossenbacher, 1996). Both phenomena can be attributed to the fact that spatial attention furthers the processing of the respective tactile stimuli, thereby making it easier to respond to them in the case of tactile targets while making it harder to ignore in the case of tactile distractors.

Turning now to the non-spatial influences of vision on the processing of tactile targets and distractors a somewhat different picture emerges. In fact, we have recently published data suggesting that vision of the stimulated body-part receiving the tactile distractor is a precondition for the processing of the distractor up to the level of response selection (see Wesslein et al., in press). This influence of vision is “distractor-specific,” as targets have always to be processed up to the level of response selection simply because participants have to respond to targets. Once again, one might consider this influence of vision on tactile distractors as some kind of attentional effect. Looking at information processing models that assume three stages of information processing (a perceptual one, a central bottleneck in which the S-R mapping is applied, and a motoric one in which the concrete response is planned; see e.g., Welford, 1952; Allport, 1989; Pashler, 1991, 1994; Spence, 2008), one may argue that vision is needed to move tactile distractors through all three stages whereas interference at the first stage (i.e., the perceptual stage) is independent of vision (note, that perceptual masking of tactile targets due to tactile distractors was independent of vision; Wesslein et al., in press). In conclusion, we would like to argue that vision influences tactile distractor processing by modulating the amount of attention that is directed to the tactile distractor. Notably, it seems as though not only spatial attention but also non-spatial attention to tactile distractors is affected by vision.

## Conflict of Interest Statement

The authors declare that the research was conducted in the absence of any commercial or financial relationships that could be construed as a potential conflict of interest.

## Footnotes

1 The “orthogonal” here refers to the fact that the dimension along which the cue varies is orthogonal to the dimension along which the targets have to be discriminated, hence ruling out a response-bias explanation for any cuing effects that may be observed (see Spence and Driver, 1997).
